# Activation of the Stress Axis and Neurochemical Alterations in Specific Brain Areas by Concentrated Ambient Particle Exposure with Concomitant Allergic Airway Disease

**DOI:** 10.1289/ehp.8619

**Published:** 2006-01-23

**Authors:** Madhu P. Sirivelu, Sheba M.J. MohanKumar, James G. Wagner, Jack R. Harkema, Puliyur S. MohanKumar

**Affiliations:** Comparative Medicine and Integrative Biology Program and Department of Pathobiology and Diagnostic Investigation, College of Veterinary Medicine, Michigan State University, East Lansing, Michigan, USA

**Keywords:** CAPs, corticosterone, hypothalamus, norepinephrine, stress axis

## Abstract

**Objective:**

Exposure to ambient particulate matter (PM) has been linked to respiratory diseases in people living in urban communities. The mechanism by which PM produces these diseases is not clear. We hypothesized that PM could act on the brain directly to stimulate the stress axis and predispose individuals to these diseases. The purpose of this study was to test if exposure to PM can affect brain areas involved in the regulation of neuroendocrine functions, especially the stress axis, and to study whether the presence of preexisting allergic airway disease aggravates the stress response.

**Design:**

Adult male rats (*n* = 8/group) with or without ovalbumin (OVA)-induced allergic airway disease were exposed to concentrated air particles containing PM with an aerodynamic diameter ≤ 2.5 μm (PM_2.5_) for 8 hr, generated from ambient air in an urban Grand Rapids, Michigan, community using a mobile air research laboratory (AirCARE 1). Control animals were exposed to normal air and were treated with saline.

**Measurements:**

A day after PM_2.5_ exposure, animals were sacrificed and the brains were removed, frozen, and sectioned. The paraventricular nucleus (PVN) and other brain nuclei were micro-dissected, and the concentrations of aminergic neurotransmitters and their metabolites were measured using high-performance liquid chromatography with electrochemical detection. Serum corticosterone levels were measured using radioimmunoassay.

**Results:**

A significant increase in the concentration (mean ± SE, pg/μg protein) of norepinephrine in the PVN was produced by exposure to concentrated ambient particles (CAPs) or OVA alone (12.45 ± 2.7 and 15.84 ± 2.8, respectively) or after sensitization with OVA (19.06 ± 3.8) compared with controls (7.98 ± 1.3; *p* < 0.05). Serum corticosterone (mean ± SE, ng/mL) was significantly elevated in the OVA + CAPs group (242.786 ± 33.315) and in the OVA-presensitized group (242.786 ± 33.315) compared with CAP exposure alone (114.55 ± 20.9). Exposure to CAPs (alone or in combination with OVA pretreatment) can activate the stress axis, and this could probably play a role in aggravating allergic airway disease.

In the past decade, numerous studies have indicated a causal association between ambient particulate matter (PM) in major metropolitan areas and potential health risks (Pope 2004). Daily death rate and hospital admissions due to pulmonary and cardiovascular problems have been shown consistently to parallel elevated PM air pollution in several epidemiologic reports (Brook 2005; Pope 2004; Samet et al. 2000; Schwartz 1999; von Klot et al. 2005).

Exposure to PM with an aerodynamic diameter ≤ 2.5 μm (PM_2.5_) has been associated with the incidence of respiratory conditions (Johnson and Graham 2005; Peel et al. 2005). Studies in various parts of the world have linked PM exposure to allergic airway diseases such as asthma (Barnett et al. 2005; D’Amato et al. 2005; Kappos et al. 2004; Trasande and Thurston 2005). PM exposure is known to cause acute respiratory events such as acute asthma exacerbations that are reflected as an increase in the symptom score as well as an increase in hospitalization and use of medication (Penttinen et al. 2001; von Klot et al. 2005). These responses are especially prevalent in populations with preexisting pulmonary disorders such as chronic bronchitis and chronic obstructive pulmonary diseases (Clarke et al. 1999). Although coarse PM (≤10 μm; PM_10_) can penetrate the human larynx and is deposited in the trachea and larger airways, finer PM_2.5_ is believed to be more toxic because of its large surface area and ease of accumulation in the alveoli of the lungs (Lippmann et al. 2005). The composition of PM is also believed to play a role in inflammatory reactions. Trace elements of anthropogenic origin such as lanthanum, vanadium, manganese, and sulfur (Morishita et al. 2004), nitric oxide (Barnett et al. 2005), tobacco smoke (Tatum and Shapiro 2005), and elemental or organic carbon from vehicle emissions (Lippmann et al. 2005; Peel et al. 2005) are all known to promote various respiratory conditions.

Although the substances associated with PM can cause inflammation on their own, the presence of PM_2.5_ in respiratory tissue can incite inflammatory reactions as well. Inhalation of PM_2.5_ results in its accumulation in the deeper portions of the lungs where it may stimulate macrophages and epithelial cells or cause an influx of neutrophils and eosinophils and promote cytokine and chemokine release (Tao et al. 2003). This may increase the permeability of the airways, and PM may enter the interstitial space where it could elicit an allergen-induced response (D’Amato et al. 2005). This would result in an increase in T-helper 2 cell (T_H_2) cytokines such as interleukin (IL)-4 and IL-13 that play an important role in the pathophysiology of allergic asthma (Marshall 2004).

Stress could be another important factor that promotes asthma. High levels of psychosocial stress are known to predict the onset of asthma and are correlated with high asthma morbidity (Sandberg et al. 2000). The elevation of stress hormones such as glucocorticoids is known not only to suppress the immune system but also to induce a shift in the T_H_1/T_H_2 cytokine balance toward a T_H_2 cytokine response, which favors the onset of asthma and allergic diseases (Marshall and Agarwal 2000). However, the effect of PM on the stress axis has not been investigated before.

We hypothesized that exposure to PM_2.5_ can cause an activation of the stress axis and that this effect would be more pronounced in animals that have existing allergic airway disease. This could be mediated through neurotransmitters such as norepinephrine (NE) and dopamine (DA) that are crucial for the stimulation of the stress axis. To test this hypothesis, we used an ovalbumin (OVA)-induced asthma model and exposed these animals to concentrated air particles (CAPs) containing PM_2.5_. The activation of the stress axis in these animals was measured by examining neurotransmitter levels in different areas of the brain related to neuroendocrine functions and by correlating them with serum corticosterone.

## Materials and Methods

Adult male Brown Norway (BN) rats 12–13 weeks old were obtained from Harlan Sprague-Dawley (Indianapolis, IN) and divided into two groups (*n* = 16). We sensitized one group to OVA by intranasally instilling a 0.5% solution of OVA in saline (150 μL/nasal passage) for 3 consecutive days. Control animals received saline intranasally. The airway sensitization of these animals was conducted in J.R.H.’s laboratory at Michigan State University. Fourteen days after the last intranasal instillation, all the rats were moved to the mobile air research laboratory (AirCARE 1; Sioutas et al. 1997) located at Calvin College (Grand Rapids, Michigan). Approximately 1 hr before the start of the inhalation exposure, rats were intranasally instilled with a 1.0% solution of OVA in saline (150 μL/nasal passage for antigen challenge) or with saline alone (controls; no antigen challenge). Rats were further divided into two groups (*n* = 8), placed in whole-body exposure chambers, and exposed to fine CAPs drawn from the local urban atmosphere or to filtered air drawn from room air (filtered air controls). We used a Harvard/U.S. Environmental Protection Agency ambient fine particle concentrator to generate the CAP exposures. The concentration of PM_2.5_ in the CAP mixture was 500 μg/m^3^. Animals were exposed to CAPs for 8 hr. Twenty-four hours after exposure, they were sacrificed by excess pentobarbital administration. The protocols were approved by the Institutional Animal Care and Use Committee at Michigan State University, and experiments were performed according to National Institutes of Health guidelines (National Institutes of Health 2002).

### Brain microdissection

At the time of sacrifice, the brain along with the olfactory bulb was quickly removed and frozen on dry ice. Serial coronal sections (300 μm thick) of the brain were obtained using a cryostat (Slee Mainz, London, UK) maintained at −10°C. The sections were transferred to precleaned microscopic slides placed on a cold stage at −10°C. Various nuclei of the hypothalamus including the paraventricular nucleus (PVN), medial preoptic area (MPA), arcuate nucleus (AN), median eminence, suprachiasmatic nucleus (SCN), and the substantia nigra (SN) were microdissected by the Palkovits’s micropunch technique (MohanKumar et al. 1998; Palkovits 1973) using a 500-μm-diameter punch with the rat brain stereotaxic atlas (Paxinos and Watson 1987) as a reference. Tissue samples were obtained bilaterally, and all the subdivisions of the nuclei were included. They were stored at −70°C until analysis for neurotransmitter concentrations using high-performance liquid chromatography with electrochemical detection (HPLC-EC).

### Neurotransmitter analysis

At the time of analysis, tissue samples were homogenized in 150 μL of 0.1 M HCLO_4_, and an aliquot of 10 μL was saved for protein analysis. The remaining sample was centrifuged briefly at 10,000×*g*, and 75 μL of the supernatant with 25 μL of internal standard (0.05 M dihydroxy benzyl amine; Sigma Chemical Co., St. Louis, MO) was injected into the HPLC system. The concentrations of NE, DA, the DA metabolite 3,4-dihydroxyphenylacetic acid (DOPAC), and the serotonin metabolite 5-hydroxyindole acetic acid (5-HIAA) were measured as described previously (MohanKumar et al. 1994, 1998). Briefly, the HPLC-EC apparatus consisted of an LC-10 AT/VP pump (Shimadzu, Columbia, MD), a phase II 5-μm ODS reverse-phase C_18_ column (Phenomenex, Torrance, CA), a glassy carbon electrode (Bioanalytical Systems, West Lafayette, IN), a model CTO-10 AT/VP column oven (Shimadzu, Columbia, MD) maintained at 37°C, and an LC-4C amperometric detector (Bioanalytical Systems, West Lafayette, IN). The data were integrated using a computer with the Class-VP chromatography Laboratory Automated Software system (version 4.2; Shimadzu).

The mobile phase was made with pyrogen-free water and contained monochloroacetic acid (14.14 g/L), sodium hydroxide (4.675 g/L), octanesulfonic acid disodium salt (0.3 g/L), EDTA (0.25 g/L), and acetonitrile (3.5%), to which tetrahydrofuran (1.4%) was added. The mobile phase was then filtered and degassed through a Milli-Q purification system (Millipore Co., Bedford, MA) and pumped at a flow rate of 1.8 mL/min. The range of the detector was 1 nA full scale, and the potential of the working electrode was 0.65 V. The sensitivity of the system was < 1 pg.

### Protein determination

Protein concentrations in the homogenates were determined using the microbicinchoninic acid assay (micro BCA; Pierce, Rockford, IL) and neurotransmitter concentrations were expressed as picograms per microgram protein.

### Radioimmunoassay

Double-antibody radioimmunoassay was used to measure corticosterone levels in the serum as described previously (Francis et al. 2000). Corticosterone standards and the I^125^-labeled corticosterone were obtained from Diagnostic Products Inc. (Los Angeles, CA). The primary and secondary antibodies were raised in our laboratory and were used at dilutions of 1:17,500 and 1:11,000 respectively. The sensitivity of the corticosterone assay was 0.2 ng/mL.

### Statistical analysis

The differences in serum corticosterone levels, neurotransmitters, and their metabolites among the various treatment groups were analyzed by one-way analysis of variance followed by post hoc Fisher’s least significant difference test.

## Results

### Paraventricular nucleus

**Figure 1:**
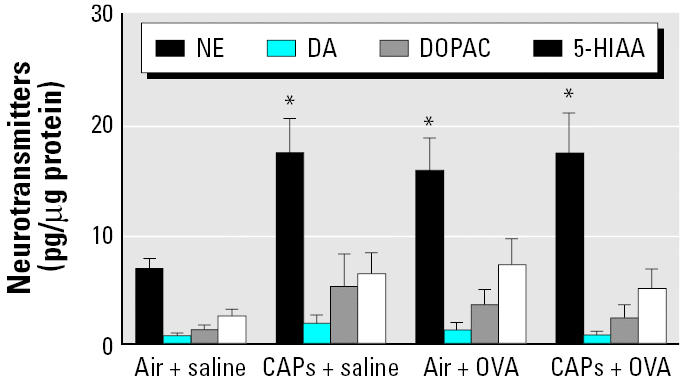
Neurotransmitter concentrations in the PVN after exposure to CAPs with or without pretreatment with OVA. Animals treated with either OVA or saline (*n* = 8 per group) were sacrificed 24 hr after an 8-hr exposure to CAPs or filtered air. *p < 0.05 compared with the air + saline group (control).

Exposure to CAPs alone or after sensitization with OVA produced significant changes in NE concentrations in the PVN (Figure 1). Exposure to CAPs alone increased NE concentrations (mean ± SE, pg/μg protein) by more than 75% (12.45 ± 2.7) compared with animals exposed to air + saline (7.98 ± 1.3; *p* < 0.05). Pretreatment with OVA increased NE levels to 15.84 ± 2.8. Exposure to CAPs after sensitization with OVA increased NE concentrations even further (19.06 ± 3.8) and produced a greater than 2-fold increase in NE levels compared with the group treated with air + saline. A trend for a decrease in DA concentrations was observed after exposure to CAPs, although it was not statistically significant. There was no significant change in the concentrations of 5-HIAA and DOPAC after exposure to CAPs.

### Medial preoptic area

**Figure 2:**
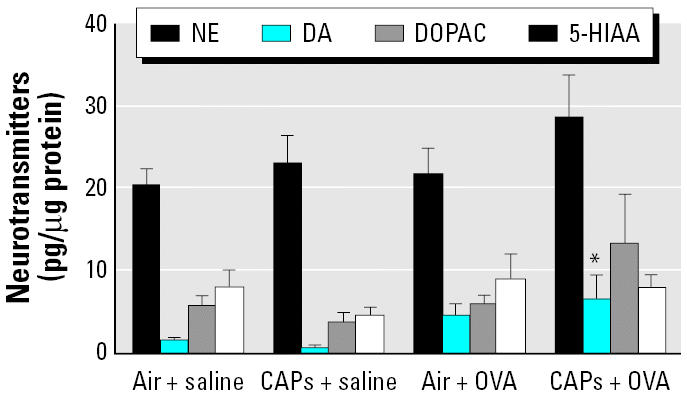
Neurotransmitter concentrations in the MPA after exposure to CAPs with or without pretreatment with OVA. Animals treated with either OVA or saline (*n* = 8 per group) were sacrificed 24 hr after an 8-hr exposure to CAPs or filtered air. *p < 0.05 compared with control animals exposed to filtered air.

In contrast to the PVN, CAP exposure resulted in an increase in the concentrations of DA in the MPA, whereas the concentrations of NE, 5-HIAA, and DOPAC remained unchanged (Figure 2). The concentration of DA in animals exposed to CAPs (0.68 ± 0.2) or OVA alone (4.51 ± 1.4) was not different from that of the controls treated with air + saline (0.93 ± 0.1). However, sensitization with OVA before CAP exposure increased DA levels significantly (6.58 ± 2.8) compared with those of the control and the CAPs + saline group (*p* < 0.05).

### Arcuate nucleus

**Figure 3:**
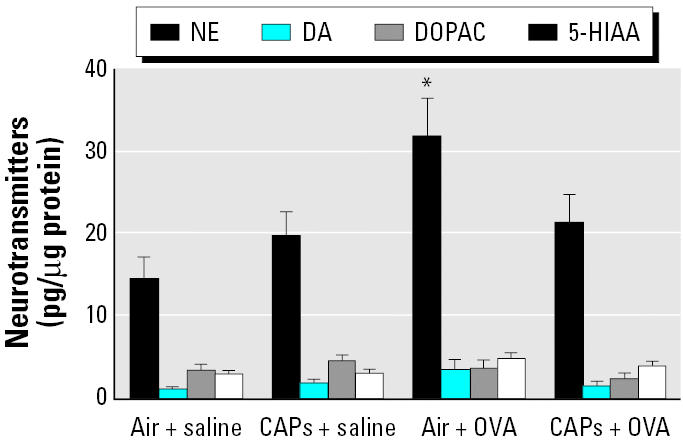
Neurotransmitter concentrations in the AN after exposure to CAPs with or without pretreatment with OVA. Animals treated with either OVA or saline (*n* = 8 per group) were sacrificed 24 hr after an 8-hr exposure to CAPs or filtered air. *p < 0.05 compared with the air + saline group (control).

Unlike the PVN and the MPA, NE levels (mean ± SE, pg/μg protein) increased in the AN after OVA sensitization (31.68 ± 4.5) compared with the air + saline controls (14.75 ± 2.4; *p* < 0.05) but not after exposure to CAPs alone (19.77 ± 2.9). Although there was a trend for an increase in the concentrations of DA, 5-HIAA, and DOPAC after CAP exposure, these changes were not statistically significant (Figure 3).

### Olfactory bulb

**Figure 4:**
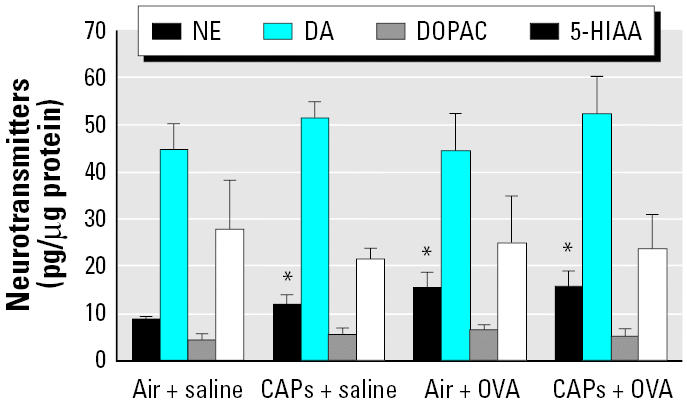
Neurotransmitter concentrations in the olfactory bulb after exposure to CAPs with or without pretreatment with OVA. Animals treated with either OVA or saline (*n* = 8 per group) were sacrificed 24 hr after an 8-hr exposure to CAPs or filtered air. *p < 0.05 compared with respective controls.

Exposure to CAPs with or without sensitization to OVA and OVA alone were all capable of increasing NE levels in the olfactory bulb (Figure 4). NE concentrations in control rats treated with air + saline were 8.8 ± 0.8 and increased significantly after exposure to CAPs (14.2 ± 0.8; *p* < 0.05). Sensitization with OVA kept NE levels elevated at 16.2 ± 1.8, and the combination of CAPs and OVA maintained it at the same level (16.1 ± 1.6). There were no significant changes in the other neurotransmitters after CAP exposure in the olfactory bulb.

### Monoamines in other areas

There were no differences in the levels of monoamines in other areas of the hypothalamus: the SCN, median eminence, ventromedial hypothalamus, or dorsomedial hypothalamus. Similarly, the SN and the cortex did not show any significant differences in neurotransmitter concentrations (Table 1).

### Serum corticosterone levels

**Figure 5:**
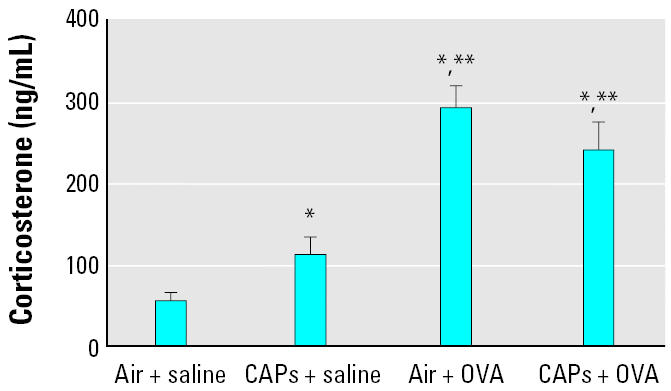
Serum corticosterone concentrations after exposure to CAPs with or without pretreatment with OVA. Animals treated with either OVA or saline (*n* = 8 per group) were sacrificed 24 hr after an 8-hr exposure to CAPs or filtered air. *p < 0.05 compared with the air + saline group. **p < 0.05 compared with the CAPs + saline group.

Levels of corticosterone in the serum (mean ± SE, ng/mL; Figure 5) paralleled the levels of NE in the PVN. Exposure to CAPs alone (114.55 ± 20.9) or after sensitization with OVA (242.786 ± 33.315) or exposure to OVA by itself (294.04 ± 26.228) produced significant increases in serum corticosterone compared with those in the control group exposed to filtered air (56.096 ± 10.2; *p* < 0.05). Moreover, exposure to CAPs after OVA sensitization, and presensitization with OVA increased serum corticosterone levels significantly compared with exposure to CAPs alone (*p* < 0.05).

## Discussion

The results of the present study indicate for the first time that exposure to CAPs can affect the concentrations of neurotransmitters in specific areas of the brain. Changes in neurotransmitters were observed in the olfactory bulb and discrete areas of the hypothalamus, such as the PVN, AN, and MPA. Neurotransmitter concentrations in other areas of the hypothalamus and the brain remained unchanged, indicating that the effects of CAPs are highly specific. Exposure to CAPs also increased circulating levels of corticosterone, paralleling the increase in NE levels in the PVN, indicating an activation of the hypothalamo–pituitary–adrenal axis or stress axis. Challenge with OVA alone produced a marked increase in stress axis activity that indicated this may be a homeostatic response to suppress inflammation. However, when OVA-sensitized animals were exposed to CAPs, stress axis activity remained elevated but no additive effect was observed. This could indicate that both OVA and CAPs act through common pathways to stimulate the stress axis.

In the present study, exposure to CAPs or OVA sensitization did not produce any change in the whole hypothalamus (data not shown). However, marked changes were observed in specific nuclei of the hypothalamus that regulate various neuroendocrine functions such as feeding, reproduction, stress, and circadian activity. Among these, the activation of the stress axis that was observed in this study is the most striking. This response could be potentially mediated through the hypothalamus. The PVN of the hypothalamus accounts for the highest concentration of corticotrophin-releasing hormone (CRH) perikarya and can effect adrenocorticotrophic hormone (ACTH) release from the pituitary upon stimulation (Szafarczyk et al. 1987). A number of neurotransmitters are known to influence CRH secretion (Muller and Nistico 1988). Among these, NE is believed to play a prominent role. The PVN receives rich noradrenergic innervation from the brainstem, and neurotoxic blockade of its noradrenergic input decreases ACTH secretion (Szafarczyk et al. 1987, 1988). Moreover, direct administration of NE into the PVN also results in an activation of the stress axis (Itoi et al. 1994). On the contrary, noradrenergic antagonists have blocked stress axis activation, emphasizing the importance of this neurotransmitter in stimulating CRH neurons (Muller and Nistico 1988). In this study, exposure to CAPs alone resulted in a robust increase in NE levels in the PVN. A similar response was observed in OVA-sensitized animals, indicating that OVA by itself can activate the stress axis. This effect was accompanied by elevated corticosterone levels in animals exposed to CAPs alone or a combination of CAPs and OVA treatment. Both NE levels in the PVN and serum corticosterone were high even in the OVA-sensitized animals upon exposure to filtered air. This observation is supported by another study in which sensitization with OVA alone activates the PVN and the central nucleus of the amygdala, another important nucleus in the stress circuitry (Costa-Pinto et al. 2005). These findings suggest that the stress axis is activated by immune challenge/allergic airway disease and that exposure to CAPs also contributes to this effect.

Our present observations indicate that NE levels are elevated in the AN as well. Noradrenergic innervation to the AN may also be involved in the stress axis. We have previously shown that NE levels in the AN are elevated after an immune stressor such as (MohanKumar et al. 1998). AN has also been implicated in autonomic functions such as IL-1 respiratory processing mediated by carotid body receptor (Banks and Harris 1988), suggesting that apart from the PVN, the AN also may be involved in stress-induced autonomic alterations.

Our previous studies involving chronic activation of the stress axis with a T_H_1 cytokine such as IL-1β for a period of 5 days indicate that NE levels in the PVN stabilize at the end of the treatment period and are not significantly different from the controls (MohanKumar et al. 2003). However, in the present study, although the animals were repeatedly exposed to OVA for 3 days, we still observed an increase in stress axis activity. This could be because OVA induces a predominantly eosinophilic response that produces a T_H_2-type immune response with increases in IL-4, IL-5, IL-10, and IL-13 (Erin et al. 2005; Kalomenidis et al. 2005). Unlike T_H_1 cytokines that directly affect the stress axis, T_H_2 cytokines, especially IL-4 and IL-13, are involved in the isotype switching from IgM to IgE, which is the antibody responsible for the generation of classical allergic reactions (Marshall 2004). However, in this study we observed an increase in stress axis activity with OVA alone. This may indicate a role for T_H_2 cytokines in stress axis stimulation, a possibility that warrants further investigation. Therefore, the nature, duration, and intensity of a specific immune response and its effect on the stress axis may depend on a delicate balance between these two classes of cytokines (Marshall 2004).

The activation of the stress axis after CAP exposure or OVA sensitization could be a homeostatic mechanism to counter the inflammatory response elicited by these paradigms. One of the mechanisms by which homeostasis is achieved could be by facilitating the elimination of tissue eosinophils through the airway lumen (Uller et al. 2006). On the other hand, corticosterone could cause a shift in the T_H_1/T_H_2 response, increasing the production of IL-4 and IL-13 that could increase the secretions in airways promoting allergic airway diseases (Erin et al. 2005; Marshall 2004).

CAP exposure has produced a marked elevation in DA concentrations in the MPA. The present study was conducted using male rats, and in males, DA in the MPA is involved in sexual motivation and copulatory behavior (Bitran et al. 1988; Pehek et al. 1988). Moreover, the MPA receives chemosensory stimuli from the olfactory bulb (Murphy and Schneider 1970), and these inputs have been implied to be obligatory for the increase in DA in the MPA during copulation (Triemstra et al. 2005). The increase in DA observed in the MPA in this study could therefore be a result of olfactory stimulation.

The olfactory bulb has been implicated in the neuroendocrine control of various autonomic activities. Stimulation of the olfactory bulb by several methods, including smoke exposure, has resulted in altered cardiovascular and respiratory parameters and increased sympathetic activity (Nakamura and Hayashida 1992). The olfactory bulb has connections to noradrenergic neurons of the brainstem (Cassell and Roberts 1991; Perez et al. 1987), especially those located in the nucleus of the solitary tract (NTS; A2 cell group) and the locus coeruleus (A6 cell group). Besides innervating the PVN, the NTS is also involved in the critical regulation of respiration (Haxhiu et al. 2005). In the NTS, a down-regulation of GABAergic inhibitory influences may lead to heightened airway responsiveness and sustained narrowing of the airways (Haxhiu et al. 2005). Because GABA (γ-aminobutyric acid) and NE are known to have a reciprocal relationship in most central functions (Muller and Nistico 1988), it is possible that the observed increase in noradrenergic activity in the olfactory bulb is related to a reduction in GABA levels, and this could translate into an altered respiratory response with the NTS as a possible relay center. Alternatively, neurons of the olfactory tract extend to the amygdala, which sends fibers to the lateral hypothalamus and the NTS besides other areas (Price 2003). This could be another potential pathway by which the olfactory lobe can affect respiration. However, further studies are needed to investigate the interconnection between the olfactory bulb and brainstem noradrenergic neurons in precipitating respiratory effects as a result of CAP exposure.

The mechanism by which CAP exposure leads to changes in neurotransmitters in the brain is unclear. The constituents and composition of the PM may be one important factor to be considered in understanding potential mechanisms. Nitric oxide, a component of PM_2.5_, can influence neurotransmitter systems (Rettori et al. 1992). Ultrafine particles in CAPs can translocate from the nasal cavity to the olfactory bulb, most likely through olfactory nerves (Oberdorster et al. 2004), where they may incite a foreign-body reaction, activating glial cells to release reactive oxygen species (Block et al. 2004; Veronesi et al. 2005). In fact, exposure to CAPs is known to increase the levels of nuclear factor κB in the brains of dogs indicating early inflammatory changes (Calderon-Garciduenas et al. 2003). It has been shown previously that lipopolysaccharide (LPS) is a constituent of CAPs as well as OVA, which was used for sensitization (Campbell et al. 2005). The same study demonstrated an increase in inflammatory cytokines, IL-1α, and tumor necrosis factor-α (TNF-α) in the brain upon exposure to CAPs. However the concentrations of LPS found were too low to stimulate IL-1 production, and the ability of LPS at these levels to stimulate IL-1 production remains questionable. LPS is shown to mediate neuroendocrine changes in the hypothalamus through IL-1 (MohanKumar et al. 1999) as well as TNF-α (Turnbull and Rivier 1998). IL-1 has been shown to produce elevations of NE in the PVN and AN (MohanKumar and Quadri 1993; MohanKumar et al. 1998) as well as elevation of ACTH and corticosterone levels (Lacosta et al. 1998). Besides increasing brain IL-1, inhaled CAPs may affect the pulmonary epithelium to cause inflammation and an increase in circulating cytokines. An increase in peripheral cytokines may also have a direct effect on brain NE levels (MohanKumar and Quadri 1993). It is possible that the activation of the stress axis by CAPs may be mediated at least in part through elevated cytokine levels.

Thus, the present study demonstrates for the first time that exposure to CAPs containing PM_2.5_ produces changes in neurotransmitter levels in specific areas of the brain and modulates neuroendocrine pathways involved in the stress axis and the autonomic control of respiratory functions. This could be one of the potential modes by which CAPs could affect the neuroendocrine and autonomic systems.

## Figures and Tables

**Table 1:** Neurotransmitter concentrations (mean ± SE, pg/μg protein) in discrete brain nuclei after exposure to CAPs.

Area	Treatment	NE	DA	DOPAC	5-HIAA
Median eminence	Air + saline	36.04 ± 8.1	44.79 ± 13.8	17.49 ± 5.0	3.75 ± 0.5
	CAPs + saline	34.43 ± 6.4	63.09 ± 27.6	18.76 ± 6.1	3.05 ± 0.7
	Air + OVA	35.40 ± 6.9	23.76 ± 2.4	6.08 ± 2.2	5.41 ± 0.8
	CAPs + OVA	24.23 ± 5.5	60.99 ± 16.5	4.15 ± 1.1	3.82 ± 0.9
SCN	Air + saline	12.42 ± 2.2	1.0 ± 0.3	3.1 ± 0.8	3.27 ± 0.5
	CAPs + saline	15.92 ± 3.1	1.2 ± 0.5	4.62 ± 0.6	5.82 ± 0.6
	Air + OVA	15.18 ± 1.2	1.3 ± 0.4	4.26 ± 0.7	6.04 ± 1.3
	CAPs + OVA	19.89 ± 3.5	2.5 ± 0.9	3.56 ± 0.7	4.29 ± 0.6
Ventromedial hypothalamus	Air + saline	11.86 ± 2.8	1.63 ± 0.8	2.11 ± 0.4	4.53 ± 0.6
	CAPs + saline	21.89 ± 2.3	1.64 ± 0.7	2.45 ± 0.4	4.83 ± 0.2
	Air + OVA	25.86 ± 4.5	1.02 ± 0.1	3.27 ± 0.6	4.80 ± 0.4
	CAPs + OVA	20.99 ± 3.0	2.02 ± 0.4	2.89 ± 0.3	5.19 ± 1.1
Dorsomedial hypothalamus	Air + saline	13.89 ± 2.4	1.35 ± 0.5	2.26 ± 0.6	5.32 ± 0.8
	CAPs + saline	13.38 ± 2.7	1.66 ± 0.7	2.16 ± 0.4	5.31 ± 0.4
	Air + OVA	13.12 ± 2.7	1.16 ± 0.2	2.39 ± 0.4	6.00 ± 0.5
	CAPs + OVA	15.47 ± 3.5	1.84 ± 0.4	2.03 ± 0.3	6.10 ± 0.6
SN	Air + saline	3.08 ± 0.5	2.24 ± 0.7	1.42 ± 0.1	5.02 ± 0.5
	CAPs + saline	4.99 ± 0.7	2.24 ± 1.0	2.30 ± 0.4	5.21 ± 0.8
	Air + OVA	5.15 ± 0.8	2.35 ± 0.3	2.70 ± 0.4	6.18 ± 0.5
	CAPs + OVA	4.71 ± 0.7	2.73 ± 0.7	2.47 ± 0.4	5.85 ± 0.5
Cortex	Air + saline	2.32 ± 0.3	0.27 ± 0.1	0.99 ± 0.1	3.02 ± 0.3
	CAPs + saline	1.86 ± 0.3	0.39 ± 0.2	1.36 ± 0.6	2.91 ± 0.4
	Air + OVA	2.06 ± 0.2	0.47 ± 0.3	1.60 ± 0.7	3.14 ± 0.3
	CAPs + OVA	1.89 ± 0.2	0.84 ± 0.4	2.23 ± 0.8	2.83 ± 0.2

Animals were treated with either OVA or saline (*n* = 8 per group) and sacrificed 24 hr after an 8-hr exposure to CAPs or filtered air.

## References

[b1-ehp0114-000870] Banks D, Harris MC (1988). Activation of hypothalamic arcuate but not paraventricular neurons following carotid body chemo-receptor stimulation in the rat. Neuroscience.

[b2-ehp0114-000870] Barnett AG, Williams GM, Schwartz J, Neller AH, Best TL, Petroeschevsky AL (2005). Air pollution and child respiratory health: a case-crossover study in Australia and New Zealand. Am J Respir Crit Care Med.

[b3-ehp0114-000870] Bitran D, Hull EM, Holmes GM, Lookingland KJ (1988). Regulation of male rat copulatory behavior by preoptic incertohypothalamic dopamine neurons. Brain Res Bull.

[b4-ehp0114-000870] Block ML, Wu X, Pei Z, Li G, Wang T, Qin L (2004). Nanometer size diesel exhaust particles are selectively toxic to dopaminergic neurons: the role of microglia, phagocytosis, and NADPH oxidase. Faseb J.

[b5-ehp0114-000870] Brook RD (2005). You are what you breathe: evidence linking air pollution and blood pressure. Curr Hypertens Rep.

[b6-ehp0114-000870] Calderon-Garciduenas L, Maronpot RR, Torres-Jardon R, Henriquez-Roldan C, Schoonhoven R, Acuna-Ayala H (2003). DNA damage in nasal and brain tissues of canines exposed to air pollutants is associated with evidence of chronic brain inflammation and neurodegeneration. Toxicol Pathol.

[b7-ehp0114-000870] Campbell A, Oldham M, Becaria A, Bondy SC, Meacher D, Sioutas C (2005). Particulate matter in polluted air may increase biomarkers of inflammation in mouse brain. Neurotoxicology.

[b8-ehp0114-000870] Cassell MD, Roberts L (1991). Ultrastructural evidence for an olfactory-autonomic pathway through the rat central amygdaloid nucleus. Neurosci Lett.

[b9-ehp0114-000870] Clarke RW, Catalano PJ, Koutrakis P, Murthy GG, Sioutas C, Paulauskis J (1999). Urban air particulate inhalation alters pulmonary function and induces pulmonary inflammation in a rodent model of chronic bronchitis. Inhal Toxicol.

[b10-ehp0114-000870] Costa-Pinto FA, Basso AS, Britto LR, Malucelli BE, Russo M (2005). Avoidance behavior and neural correlates of allergen exposure in a murine model of asthma. Brain Behav Immun.

[b11-ehp0114-000870] D’Amato G, Liccardi G, D’Amato M, Holgate S (2005). Environmental risk factors and allergic bronchial asthma. Clin Exp Allergy.

[b12-ehp0114-000870] Erin EM, Zacharasiewicz AS, Nicholson GC, Tan AJ, Higgins LA, Williams TJ (2005). Topical corticosteroid inhibits interleukin-4, -5 and -13 in nasal secretions following allergen challenge. Clin Exp Allergy.

[b13-ehp0114-000870] Francis J, MohanKumar SM, MohanKumar PS (2000). Correlations of norepinephrine release in the paraventricular nucleus with plasma corticosterone and leptin after systemic lipopolysaccharide: blockade by soluble IL-1 receptor. Brain Res.

[b14-ehp0114-000870] Haxhiu MA, Kc P, Moore CT, Acquah SS, Wilson CG, Zaidi SI (2005). Brain stem excitatory and inhibitory signaling pathways regulating bronchoconstrictive responses. J Appl Physiol.

[b15-ehp0114-000870] Itoi K, Suda T, Tozawa F, Dobashi I, Ohmori N, Sakai Y (1994). Microinjection of norepinephrine into the paraventricular nucleus of the hypothalamus stimulates corticotropin-releasing factor gene expression in conscious rats. Endocrinology.

[b16-ehp0114-000870] Johnson PR, Graham JJ (2005). Fine particulate matter national ambient air quality standards: public health impact on populations in the northeastern United States. Environ Health Perspect.

[b17-ehp0114-000870] Kalomenidis I, Stathopoulos GT, Barnette R, Guo Y, Peebles RS, Blackwell TS (2005). Eotaxin-3 and interleukin-5 pleural fluid levels are associated with pleural fluid eosinophilia in post-coronary artery bypass grafting pleural effusions. Chest.

[b18-ehp0114-000870] Kappos AD, Bruckmann P, Eikmann T, Englert N, Heinrich U, Hoppe P (2004). Health effects of particles in ambient air. Int J Hyg Environ Health.

[b19-ehp0114-000870] Lacosta S, Merali Z, Anisman H (1998). Influence of interleukin-1beta on exploratory behaviors, plasma ACTH, corticosterone, and central biogenic amines in mice. Psychopharmacology (Berl).

[b20-ehp0114-000870] Lippmann M, Gordon T, Chen LC (2005). Effects of subchronic exposures to concentrated ambient particles (CAPs) in mice. I. Introduction, objectives, and experimental plan. Inhal Toxicol.

[b21-ehp0114-000870] Marshall GD (2004). Internal and external environmental influences in allergic diseases. J Am Osteopath Assoc.

[b22-ehp0114-000870] Marshall GD, Agarwal SK (2000). Stress, immune regulation, and immunity: applications for asthma. Allergy Asthma Proc.

[b23-ehp0114-000870] MohanKumar PS, Quadri SK (1993). Systemic administration of interleukin-1 stimulates norepinephrine release in the paraventricular nucleus. Life Sci.

[b24-ehp0114-000870] MohanKumar PS, Thyagarajan S, Quadri SK (1994). Correlations of catecholamine release in the medial preoptic area with proestrous surges of luteinizing hormone and prolactin: effects of aging. Endocrinology.

[b25-ehp0114-000870] MohanKumar SM, MohanKumar PS, Quadri SK (1998). Specificity of interleukin-1beta-induced changes in monoamine concentrations in hypothalamic nuclei: blockade by interleukin-1 receptor antagonist. Brain Res Bull.

[b26-ehp0114-000870] MohanKumar SM, MohanKumar PS, Quadri SK (1999). Lipopolysaccharide-induced changes in monoamines in specific areas of the brain: blockade by interleukin-1 receptor antagonist. Brain Res.

[b27-ehp0114-000870] MohanKumar SM, Smith CL, MohanKumar PS (2003). Central adaptation to chronic administration of interleukin-1beta (IL-1beta) in rats. Brain Res Bull.

[b28-ehp0114-000870] Morishita M, Keeler G, Wagner J, Marsik F, Timm E, Dvonch J (2004). Pulmonary retention of particulate matter is associated with airway inflammation in allergic rats exposed to air pollution in urban Detroit. Inhal Toxicol.

[b29-ehp0114-000870] Muller E Nistico G 1988. Brain Messengers and the Pituitary. San Diego:Academic Press.

[b30-ehp0114-000870] Murphy MR, Schneider GE (1970). Olfactory bulb removal eliminates mating behavior in the male golden hamster. Science.

[b31-ehp0114-000870] Nakamura T, Hayashida Y (1992). Autonomic cardiovascular responses to smoke exposure in conscious rats. Am J Physiol.

[b32-ehp0114-000870] National Institutes of Health. 2002. Public Health Service Policy on Humane Care and Use of Laboratory Animals. Bethesda, MD:National Institutes of Health. Available: http://grants.nih.gov/grants/olaw/references/PHSPolicyLabAnimals.pdf [accessed 27 April 2006].

[b33-ehp0114-000870] Oberdorster G, Sharp Z, Atudorei V, Elder A, Gelein R, Kreyling W (2004). Translocation of inhaled ultrafine particles to the brain. Inhal Toxicol.

[b34-ehp0114-000870] Palkovits M (1973). Isolated removal of hypothalamic or other brain nuclei of the rat. Brain Res.

[b35-ehp0114-000870] Paxinos G Watson C 1987. The Rat Brain in Stereotaxic Co-ordinates. New York:Academic Press.

[b36-ehp0114-000870] Peel JL, Tolbert PE, Klein M, Metzger KB, Flanders WD, Todd K (2005). Ambient air pollution and respiratory emergency department visits. Epidemiology.

[b37-ehp0114-000870] Pehek EA, Warner RK, Bazzett TJ, Bitran D, Band LC, Eaton RC (1988). Microinjection of cis-flupenthixol, a dopamine antagonist, into the medial preoptic area impairs sexual behavior of male rats. Brain Res.

[b38-ehp0114-000870] Penttinen P, Timonen KL, Tiittanen P, Mirme A, Ruuskanen J, Pekkanen J (2001). Number concentration and size of particles in urban air: effects on spirometric lung function in adult asthmatic subjects. Environ Health Perspect.

[b39-ehp0114-000870] Perez H, Hernandez A, Almli CR (1987). Locus coeruleus stimulation modulates olfactory bulb evoked potentials. Brain Res Bull.

[b40-ehp0114-000870] Pope CA (2004). Air pollution and health—good news and bad. N Engl J Med.

[b41-ehp0114-000870] Price JL (2003). Comparative aspects of amygdala connectivity. Ann NY Acad Sci.

[b42-ehp0114-000870] Rettori V, Gimeno M, Lyson K, McCann SM (1992). Nitric oxide mediates norepinephrine-induced prostaglandin E2 release from the hypothalamus. Proc Natl Acad Sci USA.

[b43-ehp0114-000870] Samet JM, Dominici F, Curriero FC, Coursac I, Zeger SL (2000). Fine particulate air pollution and mortality in 20 U.S. cities, 1987–1994. N Engl J Med.

[b44-ehp0114-000870] Sandberg S, Paton JY, Ahola S, McCann DC, McGuinness D, Hillary CR (2000). The role of acute and chronic stress in asthma attacks in children. Lancet.

[b45-ehp0114-000870] Schwartz J (1999). Air pollution and hospital admissions for heart disease in eight U.S. counties. Epidemiology.

[b46-ehp0114-000870] Sioutas C, Koutrakis P, Godleski JJ, Ferguson ST, Kim CS, Burton RM (1997). Fine particle concentrators for inhalation exposures: effect of particle size and composition. J Aerosol Sci.

[b47-ehp0114-000870] Szafarczyk A, Guillaume V, Conte-Devolx B, Alonso G, Malaval F, Pares-Herbute N (1988). Central catecholaminergic system stimulates secretion of CRH at different sites. Am J Physiol.

[b48-ehp0114-000870] Szafarczyk A, Malaval F, Laurent A, Gibaud R, Assenmacher I (1987). Further evidence for a central stimulatory action of catecholamines on adrenocorticotropin release in the rat. Endocrinology.

[b49-ehp0114-000870] Tao F, Gonzalez-Flecha B, Kobzik L (2003). Reactive oxygen species in pulmonary inflammation by ambient particulates. Free Radic Biol Med.

[b50-ehp0114-000870] Tatum AJ, Shapiro GG (2005). The effects of outdoor air pollution and tobacco smoke on asthma. Immunol Allergy Clin North Am.

[b51-ehp0114-000870] Trasande L, Thurston GD (2005). The role of air pollution in asthma and other pediatric morbidities. J Allergy Clin Immunol.

[b52-ehp0114-000870] Triemstra JL, Nagatani S, Wood RI (2005). Chemosensory cues are essential for mating-induced dopamine release in MPOA of male Syrian hamsters. Neuropsychopharmacology.

[b53-ehp0114-000870] Turnbull AV, Rivier CL (1998). Intracerebroventricular passive immunization. I. The effect of intracerebroventricular administration of an antiserum to tumor necrosis factor-alpha on the plasma adrenocorticotropin response to lipopolysaccharide in rats. Endocrinology.

[b54-ehp0114-000870] Uller L, Lloyd CM, Rydell-Tormanen K, Persson CG, Erjefalt JS (2006). Effects of steroid treatment on lung CC chemokines, apoptosis and transepithelial cell clearance during development and resolution of allergic airway inflammation. Clin Exp Allergy.

[b55-ehp0114-000870] Veronesi B, Makwana O, Pooler M, Chen LC (2005). Effects of subchronic exposures to concentrated ambient particles. VII. Degeneration of dopaminergic neurons in Apo E−/− mice. Inhal Toxicol.

[b56-ehp0114-000870] von Klot S, Peters A, Aalto P, Bellander T, Berglind N, D’Ippoliti D (2005). Ambient air pollution is associated with increased risk of hospital cardiac readmissions of myocardial infarction survivors in five European cities. Circulation.

